# Replenishing the physician-scientist pipeline in the post–late bloomer era

**DOI:** 10.1172/JCI172691

**Published:** 2024-01-02

**Authors:** Daniel P. Kelly

**Affiliations:** Cardiovascular Institute and Department of Medicine, Perelman School of Medicine at the University of Pennsylvania, Philadelphia, Pennsylvania, USA.

It is my honor and privilege to deliver the AAP Presidential Address to this esteemed audience. The Presidential Address of the Spring meeting is a tradition dating back to the origins of this Society. Today, I will address a topic that is fundamental to all three linked Societies attending this meeting — *a call to action to expand and maintain the precious pool of physician-scientists*. My comments will address mentors and trainees alike.

## The early expansion of the physician-scientist pipeline: the “late-bloomer” era

I would like to begin by reminding everyone of two historic eras that spawned a rapid expansion of physician-scientists in the United States. First, under the visionary leadership of Dr. James Shannon, NIH Director from 1955 to 1968, a series of new programs was launched aimed at formally supporting the scientific training of physicians. Among these, the most impactful was the Medical Scientist Training Program (MSTP) Institutional T32 grants (1). This transformational program was designed to formally foster and support combined MD-PhD degree education at medical schools across the country. It proved to be an enormous success in terms of rapidly expanding the early-stage pool of physician-scientists. Currently, there are approximately 50 funded MSTPs and nearly as many non–NIH-funded MD-PhD programs, which together award over 600 combined degrees annually (2).

The second development was more accidental and involved the civil unrest of the late 1960s, driven in part by the Vietnam War. Idealistic medical students and residents were generally opposed to the tenets and morality of this war, leading to strategies to avoid the mandatory draft lottery following residency. Certainly, the more senior among us will remember this. One approach was to enter federally supported programs for postresidency research positions at the NIH, allowing, in essence, a legal waiver of the draft. These were highly competitive programs, so typically only the top tier of residents was selected. Notably, this often resulted in the first in-depth research exposure for these young physicians fresh out of residency training who otherwise often aspired to a clinical career. This new breed of physician-scientists has been well described, often referred to as the “Yellow Berets” or “Accidental Scientists,” as described in humorous detail by Bob Lefkowitz in his book *A Funny Thing Happened on the Way to Stockholm* (3). I will refer to this group as “late-bloomer” physician-scientists given that this started a movement that was sustained well beyond the Yellow Beret era. Many of these individuals underwent transformative career pathway shifts spawned by a newly found passion for research and, thereafter, sought to combine bench science with clinical care and teaching. The impact of this late-blooming-scientist movement at the NIH was enormous in several ways. First, this highly competitive program led to some of the brightest young physicians of their time entering biomedical research pathways, including a number of subsequent Nobel laureates (Harold Varmus, Michael Brown, Joseph Goldstein, and Bob Lefkowitz) and scientific leaders at the NIH such as Tony Fauci: our heroes! Second, this program resulted in a downstream cascade effect in subsequent years. Most took faculty positions at medical schools across the country, serving as mentors and leading by example. This started a new paradigm in which late bloomers sprung from first in-depth research experiences during their specialty fellowship training in medical schools across the country. It invigorated academic societies and expanded attendance at the annual AFCR/ASCI/AAP Spring meeting. *It was a very exciting time indeed!*

As it turns out, I was a benefactor of this era — a self-proclaimed late-bloomer physician-scientist. After finishing my medicine residency at Barnes Hospital in St. Louis, I had every intention of moving on to a clinical cardiology fellowship and a career as a clinician. My mentors and advisors, Arnie Strauss, David Kipnis, Jeff Gordon, and Burt Sobel were all directly or indirectly influenced by postgraduate “late-bloomer” research training programs at the NIH. They were inspiring and created a supportive environment that was manifest as a “culture of science” for young physicians, including myself, most of whom did not have prior in-depth experience in fundamental or applied research. For me, this led to a period of intense disruption, exhilaration, and even a touch of cognitive dissonance during my research experience as a Cardiology Fellow. But it was the “thrill of discovery,” and its potential impact on biomedicine, that led to my seeing the light and choosing a career pathway direction that ultimately led to the launching of an independent research program. Yes, it was risky, but also so exciting that I did not think about turning back.

**Figure 1:**
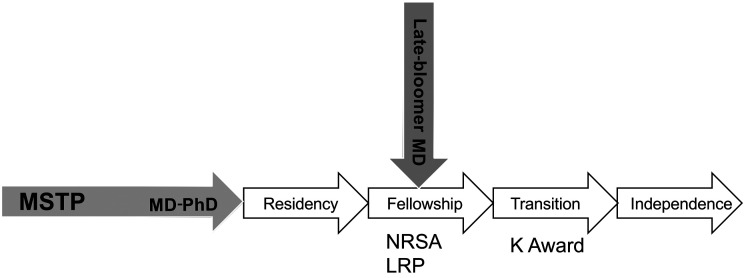
The physician-scientist pipeline.

These two converging developments defined the central pipeline that markedly expanded the cohort of physician-scientists in the United States (Figure 1). However, this remarkable swell of the physician-scientist pool required adjustments. There was increasing recognition that this rapid expansion could not be sustained unless additional support mechanisms were put in place. Indeed, Jim Wyngaarden’s AAP Presidential Address in 1979 described the physician-scientist as an “endangered species” (4). With such calls to action, a movement took place to help sustain the newly formed pipeline, triggering the NIH to develop new initiatives that invested in physician-scientist training and the transition to independence, including NIH-sponsored NRSA, K Award, and loan repayment programs. In addition, several innovative programs supported by private entities such as the Howard Hughes Medical Institute (HHMI) and Lucille Markey Foundation Scholars Programs also emerged providing support during and following graduate medical education. *I submit to you that lobbying by societies such as the AAP had a major impact during this period. At this point, and for a number of years thereafter, we were truly on a roll!*

## New challenges affecting the physician-scientist pipeline

As you all know, new challenges have emerged. This was formally described by the report of the 2014 NIH Physician-Scientist Workforce Working Group (5), in which a number of our Society members participated. The report outlined troubling statistics indicating that our physician-scientist pipeline was becoming constricted, a problem that has persisted and grown to the current time. The report concluded that (a) we are witnessing a progressive decrease in MD (single-degree) scientists; (b) increasing dropout of dual-degree physician-scientists after residency and K Award to independence; (c) the physician-scientist workforce has been aging for over a decade without commensurate new infusion. Over the past 10 years, the number of physician-scientists in their 40s has decreased from approximately 7,000 to approximately 3,800, while the number in their sixth to eighth decades continues to increase; and (d) the physician-scientist workforce lacks gender and racial diversity equity. It has been concluded that to maintain the physician-scientist workforce at its current size, an estimated approximately 1,000 physician-scientists must enter the workforce annually (6). With approximately 20% dropout, an estimated 1,200 are needed. Currently, fewer than 700 MSTP trainees enter medical school each year.

What are the drivers of this problem? Much has been written about this topic and a number of contributory factors have been identified, including, but not limited to, the following: (a) Time for clinical subspecialty training has increased, as has its intensity. MD-PhD training adds time. The average age when physician-scientists begin their career has been rising, making this pathway increasingly less attractive. (b) Medical school debt is increasing. (c) Work-life imbalance. (d) And importantly, risk-taking behavior has dramatically changed, especially during the postresidency period. Engaging in a first in-depth research experience following medical school as late bloomers is now considered a highly risky proposition.

In other words, I would argue, the traditional late-bloomer era as we knew it has ended! *We need new solutions!*

## Solutions: Early exposure to the “thrill of discovery” and other calls to action

**Figure 2:**
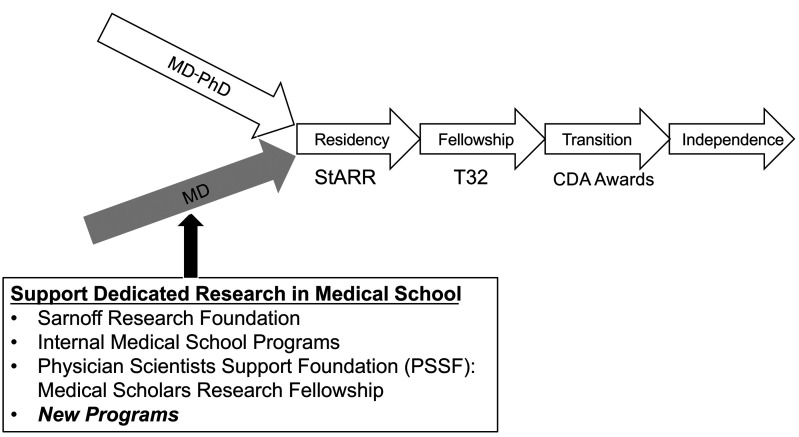
Replenishing the MD pipeline in the post–late bloomer era.

What can we do to address this impending crisis? I would like to suggest a two-pronged strategy:

*Replenish the MD pipeline in the post–late bloomer era.* We cannot simply increase the MSTP pool — doubling the number of MD-PhD trainees is neither feasible nor financially viable. We should increase the pool of single-degree physician-scientists by tapping into the far larger group of over approximately 20,000 MD-only medical students who graduate each year (Figure 2). This problem has been compounded by the “sundowning” of the HHMI Medical Research Fellows Program. We need to reactivate the excitement and impactful research conducted by the historic “late bloomers,” but at an earlier stage. Let us not underestimate the enormous impact our MD-only scientists have made (historically). The vast majority of Nobel laureate AAP members were MD-only scientists. However, this requires programmatic financial support to expand research opportunities prior to and during medical school. New avenues of support for research during medical school akin to the HHMI and related programs should be implemented. But the new programs must be designed for longitudinal success rather than simply a gap year that is used to compete for prestigious residencies — a major failing of the earlier programs! Successful models should incorporate *longitudinal mentoring* and *peer networking*. A great example is the Sarnoff Fellowship Program (7) focused on cardiovascular research that provides longitudinal mentoring and has established a peer network of emerging physician-scientists. But we need more programs. One such emerging program, recently launched by Bob Lefkowitz and colleagues, is supported by the newly developed Physician Scientist Support Foundation (https://www.thepssf.org/). This program provides not only a year of supported dedicated research during medical school, but also seeks to establish a community of like-minded peers and mentor-advisors aimed at navigating the subsequent years — akin to the Sarnoff model. Special emphasis is given to underrepresented minority students. Programs such as this need to be supported so that we can increase the pipeline of single-degree early bloomers. Think back to your own formative trainee years. We need to ensure that students are exposed to the thrill of discovery, as occurred for many of the late bloomers but *at a much earlier stage, prior to differentiation!* And we must also bolster the underrepresented minority pipeline. I would argue that we need more programs targeting high school or early undergraduate stages, particularly for this group.

**Figure 3:**
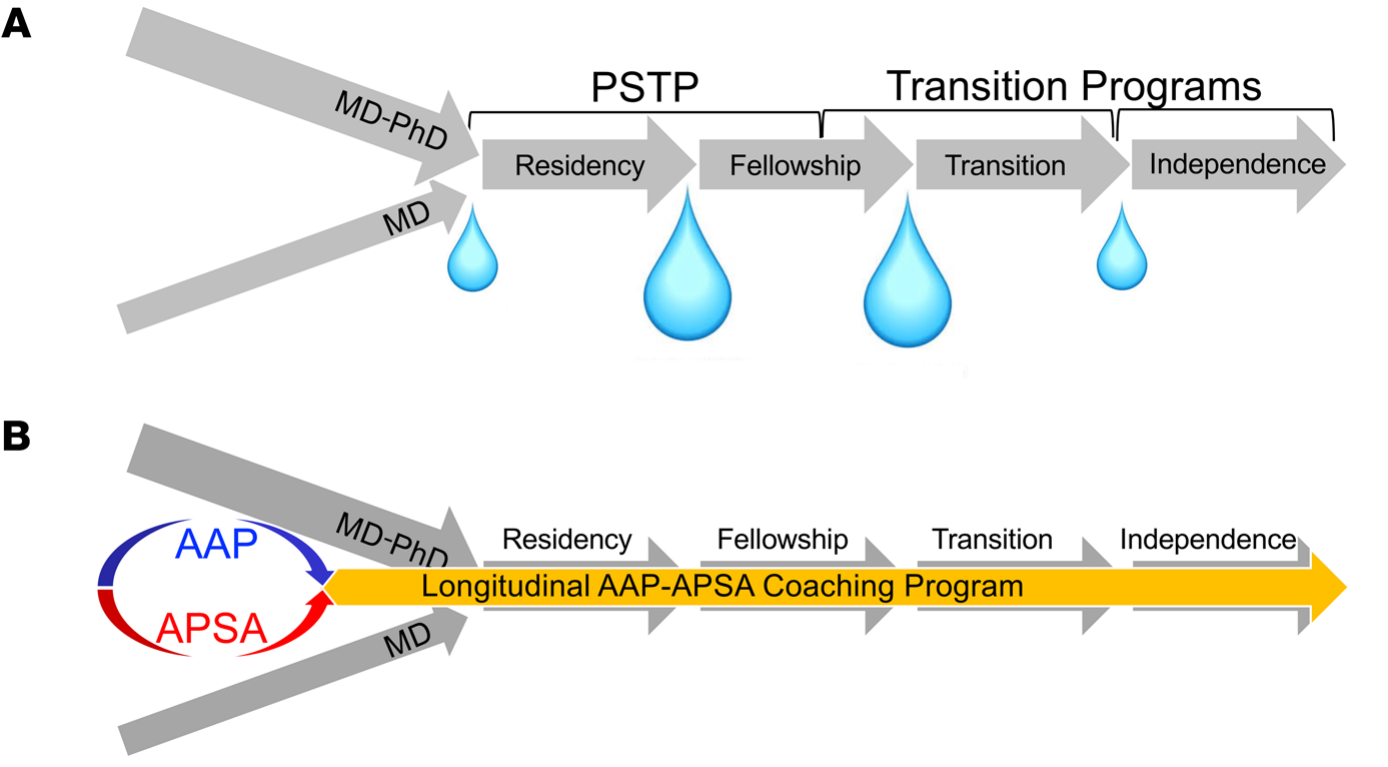
A solution for a leaky pipeline. (**A**) Physician-scientist dropout during the postgraduate period. (**B**) The AAP-APSA Longitudinal Coaching Program.

*Stop the leakage.* The second critical component of the proposed strategy is focused on the postgraduate period. Simply expanding the pipeline is not the full solution given the extraordinary dropout or “leakage” we are witnessing during the postgraduate years (Figure 3A). This is especially problematic for the dual-degree pathway in which there is heavy investment. We should reduce the timeline to independence and to address the financial stress related to debt/financial burden compared with clinical track colleagues. *We need to accelerate and finance*. Newer initiatives have clearly helped in this regard, including the Physician Scientist Training Programs (PSTP). Indeed, PSTP and MSTP leaders meet regularly, including at this meeting, to assess outcomes and establish best practices, which is laudable. Strong consideration should be given to extending PSTP programs to all specialties and subspecialties. And perhaps the NIH K99 Award–type mechanism should be established for physician scientists? But we must also institute acceleration/financial support programs during the postresidency phase — a critical transition period. An example at our institution is the Penn Measey Scholars in Molecular Medicine program (https://www.molmedscholars.org/).

Finally, I would like to introduce an initiative I am particularly excited about that addresses what I consider to be the most important unmet need — *mentorship continuity*. Arguably, this is our biggest challenge. We must provide consistent support, advice, guidance, and encouragement throughout the duration of the training and transition periods. To this end, in partnership with APSA, we propose the AAP/APSA Longitudinal Coaching Program (Figure 3B). This approach will match single- and dual-degree medical student APSA members with AAP mentor-coaches who will serve as personalized career advisors for the duration of training, from medical school to independence. Features will include (a) initial matching of coaches and trainees at the Spring meeting, starting with the APSA poster session. Indeed, a pilot approach was launched today, matching 12 AAP Council mentors with 50 APSA medical students; (b) additional perks and incentives could include expanding Trainee Travel Awards for this meeting; (c) regular interaction between coaches and mentees via virtual meetings and when possible face-to-face interactions. The interactions should continue during the postgraduate training period; and (d) ultimately, we hope that a network of participants will be established and enhanced by interactions through APSA membership.

I hope you will respond to this call to action and join the cause. Serve as lobbyists and leaders at your respective institutions. Watch for the opportunity to get involved in the emerging AAP/APSA Coaching Program. *Help create a new generation of early bloomers!*

In closing, I would like to acknowledge individuals that have truly enabled my role in serving the AAP over the past year. It has been a privilege to work with a superb and fun AAP Council. I greatly look forward to inducting 70 amazing new AAP members at our banquet this evening. It was a real honor and privilege to work with the leaders of ASCI and APSA, Sohail Tavazoie and Yentli Soto-Albrecht, respectively. Thanks to past AAP Presidents Mitch Lazar and Beth McNally for support and advice. I am very grateful to AAP Executive Director, Lori Ennis, for always steering the ship!

## Version 1 01/02/2024

Electronic publication
